# A Small Database with an Adaptive Data Selection Method for Solder Joint Fatigue Life Prediction in Advanced Packaging

**DOI:** 10.3390/ma17164091

**Published:** 2024-08-17

**Authors:** Qinghua Su, Cadmus Yuan, Kuo-Ning Chiang

**Affiliations:** 1 Department of Power Mechanical Engineering, National Tsing Hua University, Hsinchu City 30013, Taiwan; 0967356474shq@gmail.com; 2 Department of Mechanical and Computer-aided Engineering, Feng Chia University, Taichung 407102, Taiwan; cayuan@fcu.edu.tw

**Keywords:** advanced packaging, life prediction, adaptive sampling, small data, machine learning, ensemble learning, AI-assisted design of simulation (AI-DoS)

## Abstract

There has always been high interest in predicting the solder joint fatigue life in advanced packaging with high accuracy and efficiency. Artificial Intelligence Plus (AI+) is becoming increasingly popular as computational facilities continue to develop. This study will introduce machine learning (a core component of AI). With machine learning, metamodels that approximate the attributes of systems or functions are created to predict the fatigue life of advanced packaging. However, the prediction ability is highly dependent on the size and distribution of the training data. Increasing the amount of training data is the most intuitive approach to improve prediction performance, but this implies a higher computational cost. In this research, the adaptive sampling methods are applied to build the machine learning model with a small dataset sampled from an existing database. The performance of the model will be visualized using predefined criteria. Moreover, ensemble learning can be used to improve the performance of AI models after they have been fully trained.

## 1. Introduction

As the demand for electronic products continues to rise, electronic packaging gradually moves towards miniaturization and high density. Wafer-level chip scale packaging (WLCSP) offers a significant advantage in effectively reducing the package size. Analyzing solder ball reliability is crucial for assessing the long-term performance of WLCSP. The difference in the coefficient of thermal expansion (CTE) between various materials is a major factor that affects the reliability of WLCSP. In the accelerated thermal cycling test (ATCT), the failure mainly happens at the corner of the solder ball with the greatest Distance to Neutral Point (DNP) [1].

**Figure 1:**
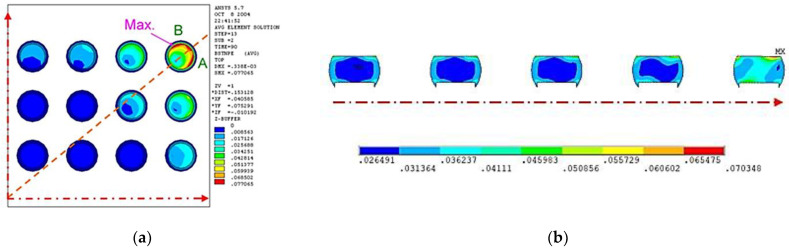
The simulation results of FEM. (**a**) 3D FEM model; (**b**) 2D FEM model.

Although experiments can provide reliable results, they require a significant amount of time, are costly, and are environmentally unfriendly; therefore, the design-by-experiment approach is not suitable for electronic packaging. In order to reduce ATCT experiment numbers and complete the initial design of packaging structures, finite element analysis (FEA) is generally used. A 3D finite element model of a wafer-level chip scale package (WLCSP) was developed by Liu et al. [2]. The incremental value of equivalent plastic strain for each cycle was calculated. The incremental value that tends to stabilize was incorporated into the Coffin–Manson equation to predict solder ball reliability, and the simulation results were close to those obtained in experiments. As a result, 3D finite element simulation can be time-consuming and involves many detailed models. According to Tsou [3], for symmetric packaging structures, a 2D finite element model can also match the experiment result by adjusting the mesh size of key regions, and the simulation time can be significantly reduced. The simulation results are shown in Figure 1. The use of simulations to replace experiments has many benefits, but it also presents challenges: the development of an effective simulation process requires expertise in domain knowledge and finite element theory, and the results obtained by different researchers may differ.

**Figure 2:**
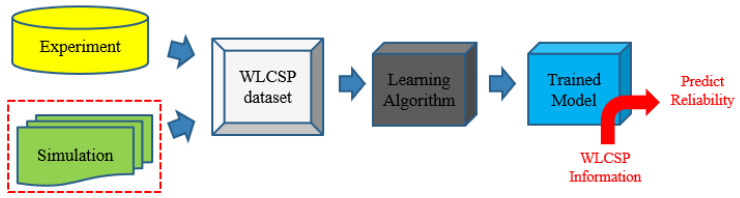
The workflow of AI-assisted Design of Simulation.

One effective approach to address the abovementioned difficulties is introducing machine learning. Due to the nonlinear modeling capabilities of the AI models, they can capture the response of the FEM with high accuracy. Hence, a well-trained AI model enables the researchers to obtain the desired results quickly. Figure 2 shows the workflow called AI-assisted Design of Simulation [4]. This chart shows that both experiments and simulations can contribute to building the dataset for WLCSP. As many simulation results need to be generated for AI training, the 3D finite element model is too time-consuming and will not be as suitable as a 2D WLCSP model. Validated 2D models are the basis for generating our raw database, and the details of this model will be presented in the sections below. Once the dataset has been validated, the learning algorithm will be used to obtain the trained model. As soon as the trained model has been obtained, the designer can enter the information about the structure of the package, and the reliability life of the WLCSP can be immediately determined.

**Figure 3:**
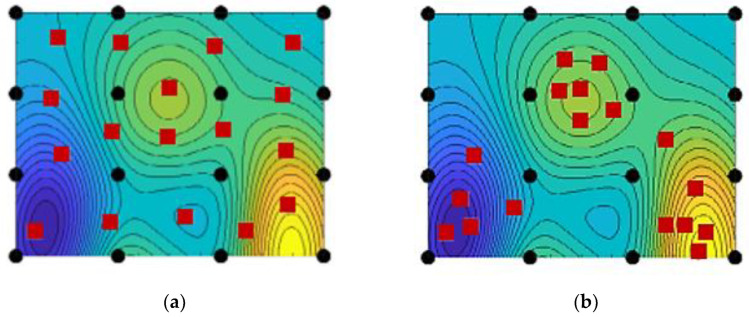
Different sampling techniques [7]. (**a**) Space-filling design; (**b**) Adaptive design. The black dots represent the initial sample points, while the red squares indicate the subsequent sample points.

The key factors of the training dataset, including the quality and quantity, directly impact the AI model’s performance [5]. As soon as the feature space of the AI model has been determined, there are generally two strategies for sampling data: the spacing-filling method and the adaptive method. Figure 3 illustrates a bounded 2D feature space. The spacing-filling technique in this figure ensures that the distribution of data points is always uniform. Furthermore, adaptive sampling techniques position a certain proportion of data points in the high-interest area. If the cost of data acquisition is not high, the spacing-filling design is the first choice to sample as many data points as possible to fill the entire feature space. Using a large training dataset can significantly improve the performance of AI models, as demonstrated quite intuitively in the previously published results of our lab [6]. Since future work will involve more design parameters, relying only on spacing-filling techniques will require an exponentially increasing number of data points to stabilize the prediction performance of AI models in the future. It is therefore important to investigate small data training based on adaptive sampling.

Markus’s work [8] demonstrated significant improvement in machine learning training results by using adaptive sampling techniques to generate data. With the aim of reducing training data without significantly affecting training results, this study will also utilize adaptive sampling techniques.

Additionally, the choice of machine learning algorithms is crucial. Referring to the process depicted in Figure 2, adaptive design is incorporated into the data generation module; this research selects an artificial neural network (ANN) and introduces ensemble learning in the training algorithm module. Dong et al. [9] conducted a comprehensive review of mainstream approaches for ensemble learning. In essence, ensemble learning combines multiple weakly supervised models to achieve a stronger and more comprehensive supervised model. As an extra machine learning strategy, this research will focus on data generation and not delve extensively into ensemble learning.

In summary, to reduce data sampling costs and model training time, this research will explore the feasibility of accurately predicting the reliability of advanced packaging using a small dataset. In fact, small data analysis has been applied across various fields. Izonin et al. [10] improved the performance of small data analysis in the field of biomedical engineering using improved neural network techniques. Zhang [11] achieved precise prediction of the target problems in the field of materials science by incorporating prior knowledge and using Kernel Ridge Regression (KRR) in cases with small datasets. These studies often focus on optimizing learning algorithms due to the uncontrollable nature of data acquisition. This paper, however, focuses on data sampling techniques because it uses simulation to generate datasets. This study will be optimized based on the process shown in Figure 2. Adaptive sampling techniques will be used to establish the dataset for WLCSP. The learning algorithm will consistently use ANNs, and the trained models will be ensemble models to predict the final reliability.

## 2. Fundamental Theory

### 2.1. Coffin–Manson Model

The Coffin–Manson equation [12] is an empirical strain-based method for predicting the fatigue life of packaging structures. Its expression is as follows:

(1) $$N_{f}=\alpha {\left({\Delta \varepsilon }_{eq}^{pl}\right)}^{\phi },$$

where $N_{f}$ is the mean time to failure (MTTF) cycles. α and ϕ are empirical constants, typically obtained through curve fitting. ${\Delta \varepsilon }_{eq}^{pl}$ represents the incremental equivalent plastic strain. It can be defined as follows [13]:

(2) $$∆\varepsilon_{eq}^{pl}=\frac{\sqrt{2}}{3}\sqrt{{\left(∆\varepsilon_{x}^{pl}-∆\varepsilon_{y}^{pl}\right)}^{2}+{\left(∆\varepsilon_{y}^{pl}-∆\varepsilon_{z}^{pl}\right)}^{2}+{\left(∆\varepsilon_{z}^{pl}-∆\varepsilon_{x}^{pl}\right)}^{2}+\frac{3}{2}∆\gamma^{pl}},$$

(3) $${∆\gamma^{pl}}^{2}=∆{\gamma_{xy}^{pl}}^{2}+∆{\gamma_{yz}^{pl}}^{2}+∆{\gamma_{xz}^{pl}}^{2},$$

where $∆\varepsilon_{x}^{pl}$, $∆\varepsilon_{y}^{pl}$, and $∆\varepsilon_{z}^{pl}$ represent the incremental plastic strain on the x, y, and z axes, respectively. $∆\gamma_{xy}^{pl}$, $∆\gamma_{yz}^{pl}$, and $∆\gamma_{xz}^{pl}$ represent the incremental shear strain on the surfaces of xy, yz, and xz. In practical calculations, finite element analysis can obtain the equivalent plastic strain of each thermal cycle, and the difference within a thermal cycle is denoted as $∆\varepsilon_{eq}^{pl}$.

### 2.2. Artificial Neural Network

Firstly, one needs to understand that machine learning can generally be divided into two categories: supervised learning and unsupervised learning. Essentially, the difference between these two methods is whether the training dataset targets are manually labeled. In this study, our task is to predict the reliability life of the packaging, since the input and output are defined, indicating that a supervised learning algorithm is indeed required. Considering both training time and predictive accuracy [14], the initial choice is the ANN algorithm to assess the quality of the small training set. Once the training set is finalized, the algorithm selection will be double-checked.

**Figure 4:**
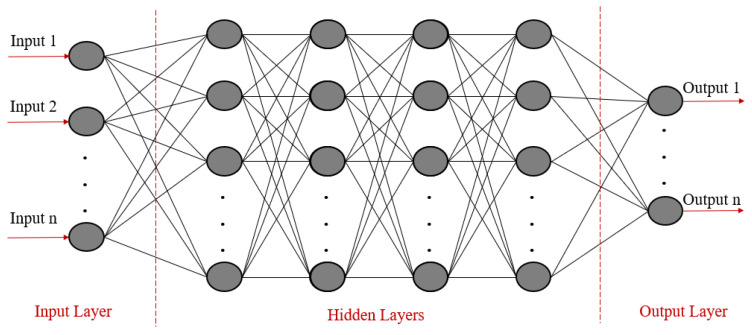
The schematic illustration of an ANN.

An ANN, proposed by McCulloch and Pitts [15], simulates how human neurons transmit information to connect inputs and outputs. The schematic illustration of its structure is shown in Figure 4. The main components include input, hidden, and output layers [16]. The circles in Figure 4 represent the basic units of the ANN, called neurons.

**Figure 5:**
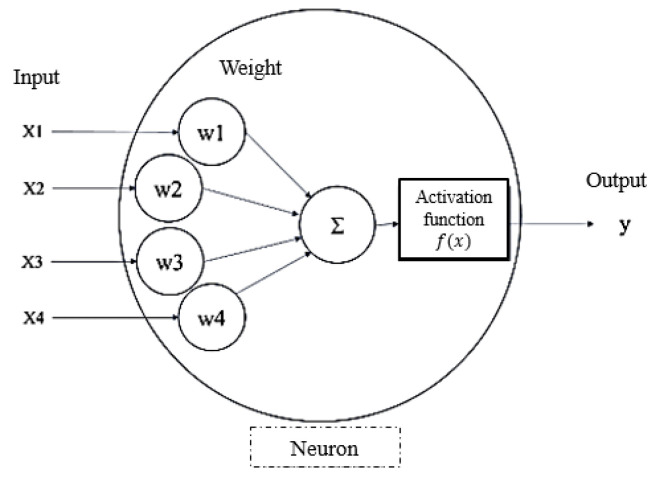
A diagram of a neuron.

The structure diagram of a single neuron with four inputs is shown in Figure 5. The inputs on the left are multiplied by the corresponding weights, and the result is fed into the specified activation function to compute the output on the right. Clearly, the values of the weights directly affect the result of the loss function. Artificial neural networks have achieved efficient iteration and automatically updated their weights using the backpropagation algorithm. This has led to ANNs becoming one of the most popular machine learning algorithms.

To perform weight updates, ANNs first need to choose an appropriate solver. This study chose two widely used solvers: Adaptive Moment Estimation (Adam) and Limited-memory Broyden–Fletcher–Goldfarb–Shanno (L-BFGS). Adam [17] is an optimization algorithm that combines the momentum method and RMSProp, offering the advantages of adaptive learning rates and momentum. L-BFGS [18] is a quasi-Newton optimization algorithm suitable for large-scale optimization problems. It achieves efficient second-order optimization by approximating the Hessian matrix.

### 2.3. K-Medoids

Like K-Means, the K-medoid algorithm [19] is a clustering algorithm. Basically, clustering is the process of dividing a dataset into different classes or clusters based on a specific criterion (such as distance), where the data points within each cluster exhibit high similarity, and the similarity between data points in different clusters is low. In accordance with the machine learning classification discussed earlier, K-medoids belong to the category of unsupervised learning.

As listed in Table 1, the essential difference between the two algorithms is that the cluster centers are actual data points in K-medoids, whereas, in K-Means, the cluster centers are computed virtual points. A clustering algorithm maximizes the dissimilarity between each cluster, and the cluster centers represent the most prominent features in the local feature space. Since K-medoid algorithms utilize actual points as cluster centers, this algorithm can be used to determine a certain number of cluster centers using an existing database as our training set. Certainly, K-Means can be used to establish a new database if starting from scratch. Despite this, rounding feature values is necessary since its cluster centers are derived from average values. In this regard, it is not recommended.

Here is a brief description of the general steps of the K-medoids algorithm:

1. Initialization: Select k data points as initial cluster centers (specify or random pick);
2. Cluster Assignment: Assign each data point to the cluster with the nearest cluster center;
3. Center Update: For each cluster, choose a new cluster center that minimizes the sum of distances to all other points in the cluster;
4. Repeat steps 2 and 3 until the cluster centers stabilize or the maximum number of iterations is reached.

**Figure 6:**
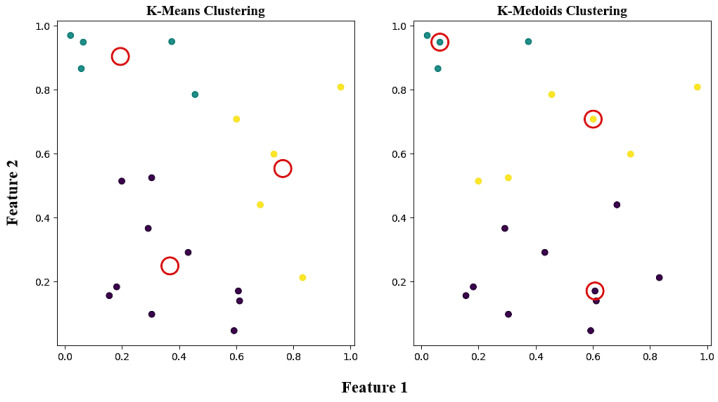
The visualization results of both algorithms. The red circles represent the cluster centers, and the three colors denote the three clusters.

Finally, the differences between the two algorithms are shown in Figure 6. As mentioned earlier, medoids are all actual sample points.

### 2.4. Ensemble Learning

Small data training becomes essential when training and acquiring data are costly. The variance of ANN-trained models is more significant when the dataset is small. In addition to using adaptive methods to optimize the distribution of training data, ensemble learning can also improve AI models’ predictive accuracy.

In ensemble learning, multiple trained models are combined to improve the performance of the model predictions. While this research will examine this method as an additional method for improving model performance, it will not be explored in depth. Generally, ensemble learning can be divided into three categories: bagging, boosting, and stacking. In this study, only the results of bagging are presented.

**Figure 7:**
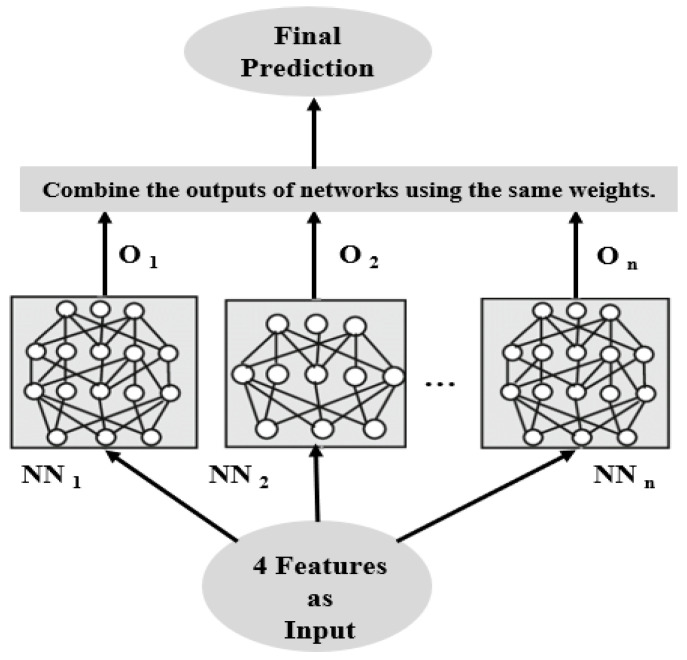
Ensemble neural networks [9].

As proposed by Breiman [20], bagging is a simple and efficient method. There are three main components to this method: training data, ensemble models, and combination. By varying the number of hidden layers and neurons, and then combining them, different ANN models can be obtained. In the case of combination, each ANN model is given equal weight, and the final prediction is the average of all ANN models. Figure 7 illustrates the schematic diagram of the structure. Generally, it is necessary to vary the data used for training each model in the ensemble when it comes to training data. In this study, ensemble learning is not the focus. To only validate the effectiveness of ensemble learning, all models will be trained using the same training set.

## 3. Validation of FEA Model

**Figure 8:**
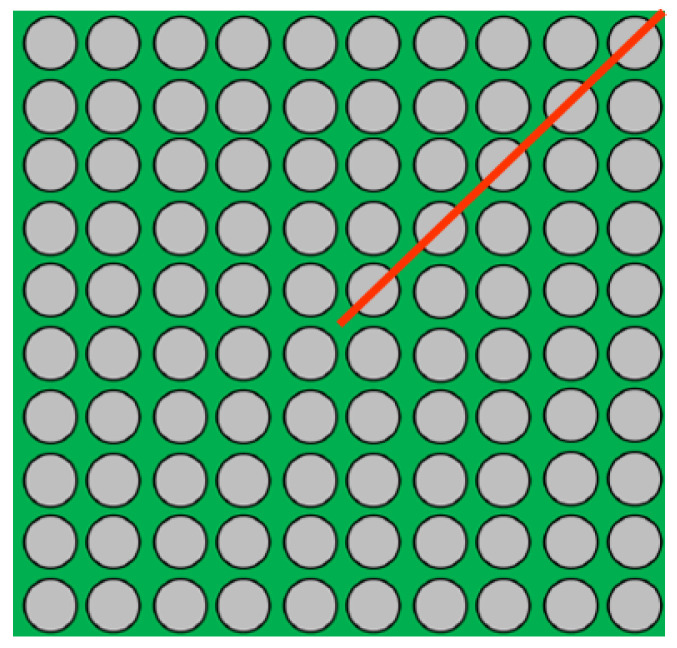
The top view of the WLCSP structure. The red line represents the half-diagonal, and the circles represent solder balls.

**Figure 9:**
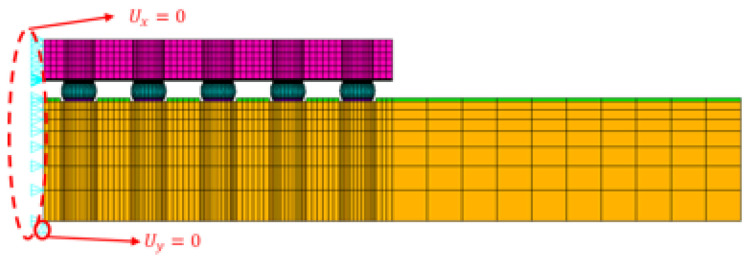
The FEA model for WLCSP.

A finite element analysis (FEA) model must be validated before constructing a simulation database. A 2D finite element model with a half-diagonal of the WLCSP will be developed in this study to reduce simulation time. Figure 8 and Figure 9 provide a schematic diagram of its structure. Figure 9 shows that the x-direction displacement at the left position is fixed to zero, while the y-direction displacement at the lower left corner of the model is also fixed to prevent rigid body motion. We set the following basic assumptions to maintain a balance between simulation efficiency and accuracy:

- It is assumed that all materials in the structure are both isotropic and homogeneous;
- The temperature is assumed to be isothermal throughout the structure;
- Residual stress is not taken into account;
- All material interfaces within the structure are assumed to be perfectly bonded.

**Figure 10:**
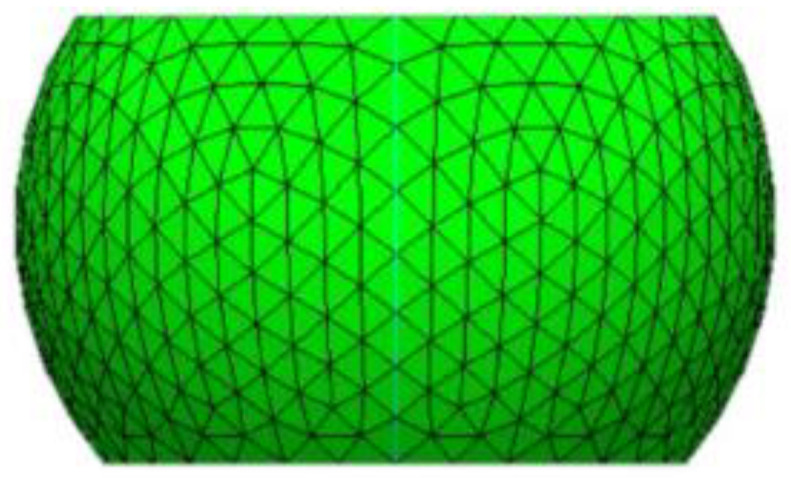
The shape of the solder ball estimated by Surface Evolver.

**Figure 11:**
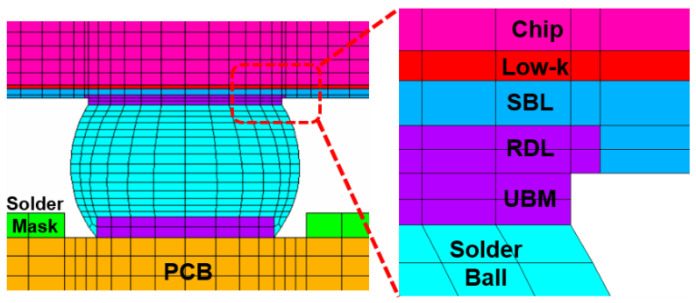
A detailed view of the FEA model.

**Figure 12:**
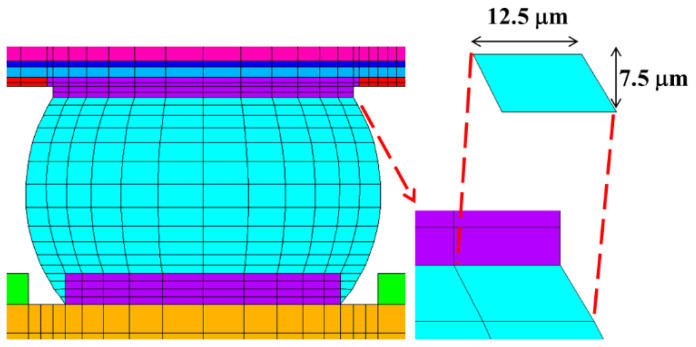
The mesh size of the key region.

FEA models will be built based on the geometric dimensions of the test vehicles. The FEA model includes the following components: silicon chip; low-k layer; stress buffer layer (SBL); under bump metallurgy (UBM); redistribution layer (RDL); printed circuit board (PCB); copper pad; solder mask; and solder ball. The Surface Evolver software V2.70 will be used to estimate the geometric dimensions of the solder balls in the structure [21]. For details, see Figure 10. It will be used to extract the coordinate information of key nodes on the solder ball for FEA. Figure 11 and Figure 12 illustrate the details of the FEA model.

**Figure 13:**
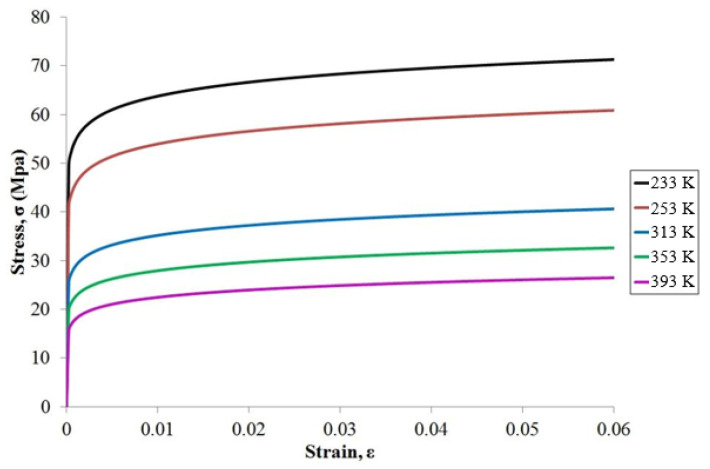
The stress–strain curve for SAC305.

As shown in Figure 12, stable simulation results can be obtained by controlling the mesh size at the top right corner of the solder ball. Generally, the first failure occurs at the top right corner of the solder ball, where maximum DNP is present. The mesh sizes Tsou [3] proposed will be adopted: 12.5 μm in the X direction and 7.5 μm in the Y direction. Except for the solder ball, all other materials are set as linear elastic. The values are shown in Table 2. In this study, the material of the solder balls is SAC305. Young’s modulus of solder SAC305 is temperature-dependent, and its mechanical properties exhibit significant nonlinearity. The Chaboche dynamic hardening model is utilized to describe its nonlinearity at different temperatures. As shown in Figure 13, the stress–strain curves for SAC305 were obtained by performing uniaxial tensile tests on the material [22]. These data will be used for curve fitting to obtain Chaboche model parameters. The formula is as follows:

(4) $$\alpha =\frac{C}{\gamma }\left(1-e^{-\gamma {\cdot \varepsilon }_{p}}\right)+\sigma_{0}$$

where $\sigma_{0}$ represents the initial yield stress. C and γ are both empirical coefficients. The parameter fitting results are shown in Table 3.

**Figure 14:**
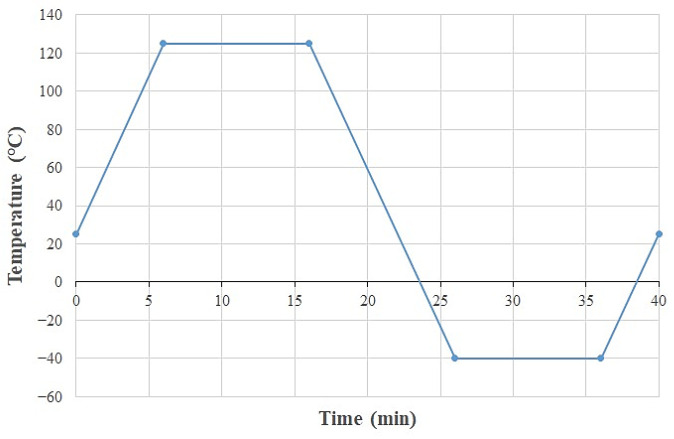
Thermal cycling temperature profile.

Thermal cycling loads are applied to the WLCSP model according to JEDEC standard Condition G, with temperatures ranging from −40 to 125 °C. The thermal cycling load remains at a dwell time of 10 min and a heating and cooling rate of 16.5 °C/min. It takes 40 min to complete one cycle. See Figure 14 for details.

Using the Coffin–Manson equation, the simulated value of packaging reliability can be obtained with incremental equivalent plastic strain. Table 4 compares the mean time to failure (MTTF) of the experiments [23,24] and simulation results for five test vehicles (TVs). This table shows that the differences between simulated and experiment values are within an acceptable range (<10%). Therefore, the fatigue life of the packages can be accurately predicted. Based on the validated FEA models, theories, material constitutive equations, solution procedures, etc., the database will be built for machine learning. To generate the database, the material parameters, boundary conditions, and temperature load are fixed. Only the geometric dimensions of the WLCSP model are changed. The AI model will be trained using the database.

## 4. Data Sampling and Model Training

To demonstrate the feasibility of small data training, this study will build the database and extract small samples from the database. The database establishment, data sampling, and model training will be described in detail.

### 4.1. Establish Database

**Figure 15:**
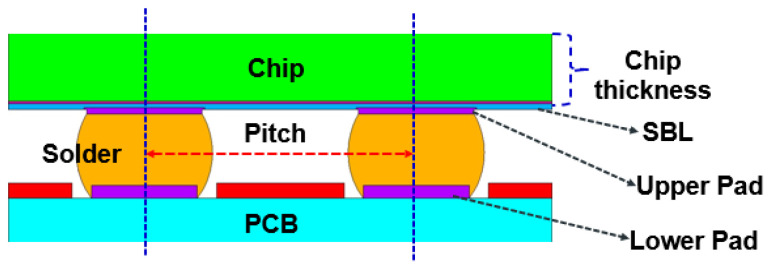
Design parameters for WLCSP structure.

Selecting features is typically the first step in establishing a database. Researchers need to choose features that are highly correlated with the predictive target [25], which often requires expert knowledge or numerical analysis. Figure 15 illustrates features highly correlated with WLCSP fatigue life based on expert knowledge. As an extension of the previous research [14], this study still uses the selection of four features. And the foundation for establishing the database is TV2. The distribution of the four features is as follows: upper pad diameter, lower pad diameter, SBL thickness, and chip thickness.

Following the concept of space-filling in previous studies, we used as many data points as possible to fill the entire feature space evenly. We were able to achieve good performance in training the AI model. The database can be obtained by determining the value boundaries of the features and selecting node values for complete permutation and combination, as shown in Table 5, Table 6, Table 7, Table 8, Table 9 and Table 10.

This is the simplest form of space-filling. In total, there are over 9000 data points. We selected a certain proportion from them as the training set, and the performance of the AI model will improve with increased training data. Previous studies did not introduce additional sampling strategies and just used random sampling. This research will introduce the adaptive sampling method that will reduce the training data while maintaining the performance of the model.

### 4.2. Data Sampling (Random Pick)

**Figure 16:**
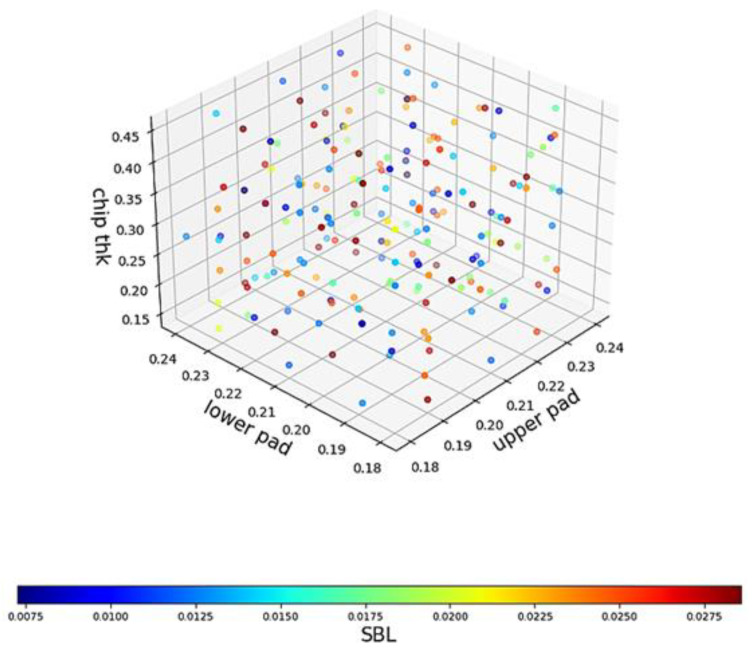
The visual distribution of the 200 training data points (random pick).

In Section 4.3, K-medoids will be used for data sampling. As a comparison group, 200 samples were randomly selected for training. The visual distribution is shown in Figure 16.

This figure’s three axes represent the upper pad diameter, lower pad diameter, and chip thickness, while the “color bar” represents the SBL thickness. This image cannot directly assess the data distribution quality, and it needs to rely on the performance of AI models to evaluate it. Two hundred data points will be used as the training set, while the remaining data points will serve as the testing set. The performance of the AI model on the testing set will be the basis for evaluation.

It is worth mentioning that in classification problems, the decision boundary is often the focal point of research. Guan [26] revealed the impact of decision boundary complexity on model generalization. To address generalization issues, a commonly used method is boundary sampling. We identified data points close to the decision boundary within the existing dataset and generated additional samples near the boundary using interpolation or other techniques [27]. Although there are no decision boundaries in regression problems, the performance of AI regression models near feature boundaries is also worth exploring. In this study, the essence of adaptive sampling lies in sampling near feature boundaries. Its impact on AI model performance will be further investigated in Section 4.3.

To assess the performance of AI models, standards need to be established, including maximum training differences, average training differences, maximum testing differences, average testing differences, the number of testing data points for a “difference > 50 cycles” and the number of testing data points for a “difference percentage > 7%”. A training difference indicates whether the model is underfitted, whereas a testing difference indicates whether it is overfitted. Using the other two standards, it is possible to determine the number of test points with inaccurate predictions intuitively. The preliminary preparation for model training has been completed so far. The ANN learning algorithm is being used with 200 training data points and over 9000 testing data points. The hyperparameter settings for the ANN are shown in Table 11.

As mentioned in Section 2.2, the learning rate of Adam is adaptive. It is a simple model with only four inputs and one output, so there are not too many tricks involved. The hyperparameter settings and the selection of data preprocessing were determined based on previous experience [14]. Here, data preprocessing specifically refers to data transformation. It is a method to adjust the range of feature values, which can optimize model performance [28]. A robust scaler is the best choice in this case. It uses quartiles for data standardization, as shown in Equation (5). Grid search is used to find the optimal combination of neuron numbers for each layer. The maximum number of iterations is set as the condition to terminate model updates.

(5) $$x^{*}=\frac{x-Q_{2}}{Q_{3}-Q_{1}}$$

Here are two sets of results, as listed in Table 12. “Neuron number” indicates the number of neurons in each hidden layer. These two models were selected from many models generated by the grid search, and they exhibit good performance in testing differences.

The data corresponding to the “Maximum difference” in the table are as follows: prediction, target, absolute difference, and percent difference. The training differences of both models indicate that they have been sufficiently trained, and there is no underfitting. Testing differences indicate that the models were not accurate in predicting unknown data. In both cases, the maximum percentage of testing errors exceeds 15%. The number of test data points with inaccurate predictions (difference ≥ 50 cycles) exceeds 200. There is no doubt that the inadequacy of the training data is a contributing factor to the poor performance of the model. The small training set obtained through random sampling needs further optimization, either by increasing its size or by improving its distribution.

Increasing the number of data points in the regions of high interest is one of the most direct methods for improving the distribution of data, and adaptive sampling refers to this method. The method relies on acquiring prior knowledge, meaning the locations of the high-interest regions must be known. As previously mentioned, boundary sampling is noteworthy but requires further validation. It is necessary to first observe whether the inaccurately predicted test points tend to cluster near the feature boundaries. A clear clustering of these test points will indicate that these clustered regions are high-interest areas.

Except for SBL thickness, the results of the other three features are very similar, with proportions close to half. The detailed results are shown in Table 13.

Among the 319 inaccurately predicted test points for Model I, 220 data points have an SBL thickness of less than 10.5 μm, accounting for a substantial portion of 70%. There are 153 data points, accounting for 48%, with the upper pad diameter at the boundary value. The situation with lower pad diameters and chip thicknesses is similar to that of the upper pad diameters. It is evidently necessary to increase the number of training data points near the feature boundaries and in regions with a small SBL thickness.

### 4.3. Data Sampling (Adaptive Method)

The results in Section 4.3 are consistent with our expectations: half of the inaccurate predictions are located at the feature boundaries. Just as data points near the decision boundary are prone to classification errors, data points near the feature boundaries also increase the risk of inaccurate predictions. Next, K-medoids will be used to perform feature boundary sampling. Since the dissimilarity between clusters, the uniformity of each cluster, and the space-filling principle are guaranteed by the clustering algorithms, the cluster centers are chosen to represent the characteristics of the clusters.

To increase training data near the feature boundaries uniformly, the feature space is split first. After comparative testing, spatial partitioning using four features versus three features has a limited impact on the final prediction performance. This study provides a focused analysis of one situation.

By dividing the upper pad, lower pad, and SBL into two sets each, and using permutations and combinations, the entire feature space can be divided into eight parts. From Table 14, in set 1, the upper and lower boundary values of the upper and lower pads are extracted. Referring to Table 13, we set 10.5 as the dividing value for the two sets of the SBL. Using K-medoids, we generated 25 cluster centers in each of the eight regions after partitioning for a total of 200 training data points.

Table 15 displays the key configuration parameters of K-medoids. “Metric” specifies the measure used to compute distances between data points, with Euclidean distance being the most widely utilized method. “Method” specifies the specific approach used for clustering, with “Alternate” being chosen based on time cost considerations. “K-medoids++” is an initialization method for cluster centers that ensures the centers are initialized with sufficient distance between them, facilitating quicker convergence to improved clustering outcomes. Set “random_state” to ensure consistency in random results. The distribution of new training data is shown in the figure below.

**Figure 17:**
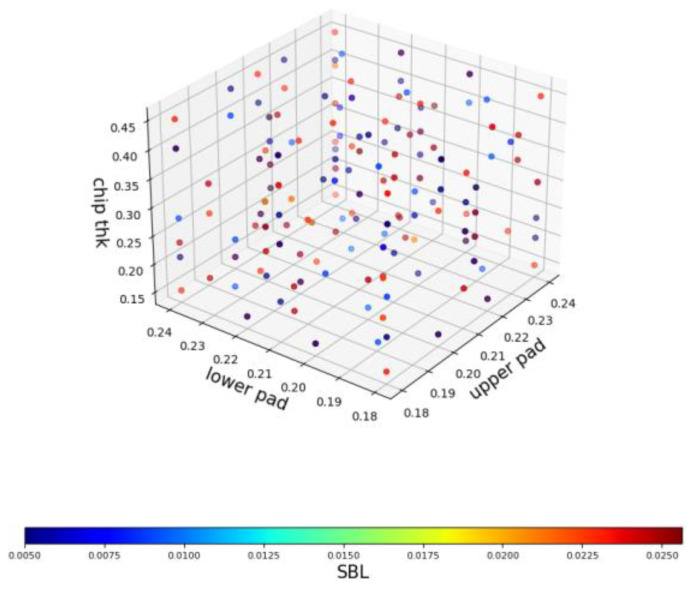
The visual distribution of new training data points (K-medoids).

Compared with Figure 16, Figure 17 shows a significant increase in the number of data points on the boundary surfaces. In addition, the number of blue data points with a small SBL thickness has increased. The AI models begin to be trained in Section 4.4 with new training data.

### 4.4. AI Model Training

Besides continuously adjusting the hyperparameters of a model of four inputs and one output, the AI model’s prediction ability relies on the data quality and quantity. Given the limited quantity, previous work improved the quality of the training dataset. The new training set will be validated in this section.

Before applying new algorithms, the selection of the ANN should be validated against other known AI models. Kou et al. [4] and Su [6] reported the rather high prediction capability of Support Vector Regression (SVR) and Kernel Ridge Regression (KRR). Table 16 compares the prediction performance of different algorithms. To reiterate the learning task, there were 200 training data points with K-medoids, 9000+ testing data points, 4 inputs, and 1 output.

All models were preprocessed using the robust scaler. In the table below, two ANN models are presented using different solvers. For ANN-1, the solver is ‘Adam’, while for ANN-2, the solver is ‘L-BFGS’. Both solvers are commonly used for ANNs. However, ‘L-BFGS’ can be more effective for small datasets [29]. The average training difference for each algorithm is small, which indicates that all algorithms have been trained successfully without underfitting. Based on the average testing difference, the ANN outperforms SVR and KRR. Therefore, the ANN will be explored in greater depth. The table below illustrates the performance of ANN models with different solvers, hidden layers, and neurons.

From Table 17, ANN-2 is the best-performing model in comparison. And it is obvious that the adjustment of important hyperparameters for the ANN has a limited impact on the average testing difference. Training a single ANN model is unable to improve performance further. On the other hand, the time cost for training on small datasets is much lower than that for training on large datasets. This is one of the advantages of training with small datasets.

Table 18 compares the prediction performance of ANN models with old and new training data. “Random pick” selected Model II, while “K-Medoids” selected ANN-2. It demonstrated that the adaptive sampling method is useful.

The new training dataset has greatly improved the performance of the AI model. The performance of the maximum testing difference has significantly improved. The number of test points with failed predictions has significantly decreased. Even though the performance is already quite good, there is room for further improvement. With the help of ensemble learning, the performance can be further improved.

### 4.5. Ensemble Learning

The ensemble learning shows high potential to improve the prediction accuracy against small datasets [30]. This study only presents some preliminary results. Voting methods, or weighted averaging, have long been effective means of reducing system variance. That is also the core idea of “bagging”. As mentioned earlier, the performance of a single ANN model is stable in testing data. Although their performance is close in numerical metrics, different ANN models often have distinct areas of inaccurate prediction.

Table 17 presents the performance of some existing ANN models from grid search. In fact, there are many ANN models that are well trained. Although individual ANN models may have limited performance, aggregating them can enhance performance. Under different hidden layers, we selected the ANN models with excellent performance on the testing set as the sub-models. All sub-models in this study have equal weights. Table 19 shows the performance results with 5 and 15 sub-models. At the same time, the final comparison results are shown in Table 20.

When new testing data are input, each sub-model will provide its own prediction, and the final result will be the average of the results from multiple sub-models. Despite having only ANN models as sub-models and not yet involving a mixture of algorithms in the ensemble, the performance of the ensemble model is still outstanding. The number of failed prediction test points has further decreased, reaching below 10. Table 20 shows the hyperparameter settings for the sub-models used, with any unmentioned hyperparameters remaining consistent with those in Table 11. To increase sub-model diversity, some trained models were added using the standard scaler. The ensemble learning of 5 sub-models uses the sub-models numbered 1–5. The final comparison results have been shown in Table 21.

Through intuitive comparison, both K-medoids and ensemble learning have the role of improving the performance of prediction.

## 5. Conclusions

Solder joint fatigue is one of the key reliability concerns for advanced electronic products, and identifying the mean time to failure of solder joints is very time-consuming and expensive. The development time will depend on the developer’s simulation/fundamental/domain knowledge and experience, and it will usually take several months to several years to determine the mean time to failure of solder joints. Applying AI-assisted Design-of-Simulation (AI-DoS) technology enables fast, accurate, consistent, and reliable prediction, as indicated in Figure 2. However, using as little data as possible to obtain a high-quality AI-trained model becomes a critical issue.

This study demonstrates the importance of training data quality and the immense potential of ensemble learning. Both core strategies of data sampling (space-filling method and adaptive method) have their merits.

When there are fewer features (inputs), utilizing the space-filling method to continuously generate data to fill the entire feature space is a good choice. The performance of the AI model will also improve as the training data increase. However, as the number of features increases, the number of data points required to effectively fill the feature space will grow exponentially. When data acquisition costs cannot be ignored, training with small data becomes meaningful.

Therefore, adaptive sampling methods have garnered significant attention in constructing proficient AI models with as few samples as possible. The challenge with adaptive methods lies in accurately identifying the areas of high interest. Clearly, the areas where the AI model’s prediction is inaccurate are the areas of high interest. In Section 4.2, the hypothesis was formulated regarding the regions of high interest (feature boundary), which were subsequently validated in later chapters. The failed prediction test points are typically concentrated at the boundaries of the feature space and in areas with a small SBL thickness. With these findings, K-medoids were used to construct new training data.

By comparison, it was found that the ANN has a clear advantage over KRR and SVR under small data training. In terms of solver selection, under small data training conditions, L-BFGS performs better than Adam. The AI models trained on the new training set show a significant improvement in prediction performance, demonstrating the adaptive method’s effectiveness.

What is more, the excellent performance of ensemble learning (bagging) also confirms the tremendous potential of this method in training with small data. The average testing difference decreases to within ten cycles, and the number of testing points with inaccurate predictions (difference ≥ 50 cycles) is reduced to fewer than 10. In future work, it is worth exploring various aspects, such as boosting and stacking with different algorithms.

On the other hand, this study involves only four features. If the number of features continues to increase, the quantity of training data is clearly not fixed. Determining how to efficiently identify the amount of training data required for a specific problem is a topic worth exploring.

## Figures and Tables

**Table 1:** Two main differences between two algorithms.

Algorithm	Cluster Center	Updated Method
K-Medoids	Actual sample point	Minimization of the sum of distances
K-Means	Virtual point	The average value of the data points

**Table 2:** Linear elastic material parameters for WLCSP.

Material	Young’s Modulus (GPa)	Poisson’s Ratio	CTE (ppm/°C)
Silicon chip	150	0.28	2.8
Cu	68.9	0.34	16.7
SBL	2	0.33	55
Low-k	10	0.16	5
Solder ball	Nonlinear	0.4	22.36
Solder mask	6.87	0.35	19
PCB	18.2	0.19	16

**Table 3:** Parameters for Chaboche model.

T (K)	$\sigma_{0}$ (GPa)	C	γ
233	47.64	8894.8	639.2
253	38.87	8573.3	660.0
313	24.06	6011.4	625.2
353	18.12	5804.2	697.7
395	14.31	4804.6	699.9

**Table 4:** The comparison of five TVs’ fatigue life.

TV	${\Delta \varepsilon }_{eq}^{pl}$	MTTF (Cycle)	Simulation (Cycle)	Difference (Cycle)	Difference (%)
1	0.0164	318	313	5	1.54%
2	0.0085	1013	982	31	3.05%
3	0.0114	587	587	0	0.00%
4	0.0096	876	804	72	8.20%
5	0.0091	904	885	19	2.12%

**Table 5:** Feature values of 256 data points.

Features	Feature Values
Upper Pad Dia. (Unit: mm)	0.18, 0.2, 0.22, 0.24
Lower Pad Dia. (Unit: mm)	0.18, 0.2, 0.22, 0.24
Chip Thickness (Unit: mm)	0.15, 0.25, 0.35, 0.45
SBL Thickness (Unit: μm)	5, 14.17, 23.33, 32.5

**Table 6:** Feature values of 625 data points.

Features	Feature Values
Upper Pad Dia. (Unit: mm)	0.18, 0.195, 0.21, 0.225, 0.24
Lower Pad Dia. (Unit: mm)	0.18, 0.195, 0.21, 0.225, 0.24
Chip Thickness (Unit: mm)	0.15, 0.225, 0.300, 0.375, 0.45
SBL Thickness (Unit: μm)	5, 11.88, 18.75, 25.63, 32.5

**Table 7:** Feature values of 1296 data points.

Features	Feature Values
Upper Pad Dia. (Unit: mm)	0.18, 0.192, 0.204, 0.216, 0.228, 0.24
Lower Pad Dia. (Unit: mm)	0.18, 0.192, 0.204, 0.216, 0.228, 0.24
Chip Thickness (Unit: mm)	0.15, 0.21, 0.27, 0.33, 0.39, 0.45
SBL Thickness (Unit: μm)	5, 10.5, 16, 21.5, 27, 32.5

**Table 8:** Feature values of 2401 data points.

Features	Feature Values
Upper Pad Dia. (Unit: mm)	0.18, 0.19, 0.2, 0.21, 0.22, 0.23, 0.24
Lower Pad Dia. (Unit: mm)	0.18, 0.19, 0.2, 0.21, 0.22, 0.23, 0.24
Chip Thickness (Unit: mm)	0.15, 0.2, 0.25, 0.3, 0.35, 0.4, 0.45
SBL Thickness (Unit: μm)	5, 9.58, 14.17, 18.75, 23.33, 27.92, 32.5

**Table 9:** Feature values of 4096 data points.

Features	Feature Values
Upper Pad Dia. (Unit: mm)	0.18, 0.189, 0.197, 0.206, 0.214, 0.223, 0.231, 0.24
Lower Pad Dia. (Unit: mm)	0.18, 0.189, 0.197, 0.206, 0.214, 0.223, 0.231, 0.24
Chip Thickness (Unit: mm)	0.15, 0.189, 0.197, 0.206, 0.214, 0.223, 0.231, 0.24
SBL Thickness (Unit: μm)	5, 9.58, 14.17, 18.75, 23.33, 27.92, 32.5

**Table 10:** Feature values of new 1296 data points.

Features	Feature Values
Upper Pad Dia. (Unit: mm)	0.184, 0.194, 0.205, 0.219, 0.226, 0.234
Lower Pad Dia. (Unit: mm)	0.184, 0.194, 0.205, 0.219, 0.226, 0.234
Chip Thickness (Unit: mm)	0.174, 0.221, 0.289, 0.341, 0.379, 0.426
SBL Thickness (Unit: μm)	7.25, 12.55, 17.95, 22.65, 27.35, 30.35

**Table 11:** The hyperparameters of the ANN.

Hyperparameter	Setting
Preprocessing	Robust scaler
Activation function	Relu
Solver	Adam/L-BFGS
Learning rate	Adaptive
Initial learning rate	0.001
Hidden layers	4
Neuron number	Grid search
Max_iter	5000

**Table 12:** The performance of two ANN models.

Item	Model I	Model II
Neuron number	64-72-88-88	88-72-72-56
Maximum training difference	1413/1437/24 (1.7%)	0 (0)
Average training difference	6.6 (0.65%)	0 (0)
Maximum testing difference	1005/863/142 (16.5%)	755/648/107 (16.5%)
Average testing difference	14.6 (1.5%)	12.4 (1.3%)
≥50 cycles	319	205
≥7%	190	115
CPU time (s)	1.8	7.2

**Table 13:** Data distribution of inaccurately predicted test points.

Data Distribution	Model I	Model II
SBL thickness (<10.5 μm)	220/319/70%	150/205/73%
Upper pad dia. (0.18 or 0.24 mm)	153/319/48%	113/205/55%

**Table 14:** The partitioning of the feature values.

Feature	Value Range of Set 1	Value Range of Set 2
Upper Pad Dia. (Unit: mm)	[0.18, 0.19] and [0.23, 0.24]	(0.19, 0.23)
Lower Pad Dia. (Unit: mm)	[0.18, 0.19] and [0.23, 0.24]	(0.19, 0.23)
Chip Thickness (Unit: mm)	——	——
SBL Thickness (Unit: μm)	[5, 10.5]	(10.5, 32.5]

——: Not involved in the split.

**Table 15:** The hyperparameters of K-medoids.

Hyperparameter	Setting
N_clusters	25
Metric	Euclidean
Method	Alternate
Init	K-medoids++
Random_state	1
Max_iter	500

**Table 16:** The performance results of different algorithms.

Performance	ANN-1	ANN-2	KRR	SVR
Maximum training difference	26 (3.8%)	0 (0)	30 (2.6%)	65 (5.5%)
Average training difference	4.9 (0.5%)	0 (0)	4.9 (0.5%)	6.8 (0.7%)
Maximum testing difference	95 (5.5%)	76 (6.8%)	231 (14.1%)	207 (11.9%)
Average testing difference	11.9 (1.2%)	10.5 (1.0%)	18.2 (1.6%)	19.0 (1.7%)
CPU time (s)	2.3	4.6	0.1	0.1

**Table 17:** The performance results of different ANN models.

Item	ANN-1	ANN-2	ANN-3	ANN-4	ANN-5	ANN-6
Solver	Adam	LBFGS	Adam	LBFGS	Adam	LBFGS
Neuron number	72-88-40-48	72-24-8-80	76-28-24	76-80-56	72-98	56-42
Maximum training difference	26 (3.8%)	0 (0)	44 (6.4%)	0 (0)	56 (5.5%)	18 (1.6%)
Average training difference	4.9 (0.5%)	0 (0)	8.1 (0.9%)	0 (0)	8.4 (0.9%)	2.8 (0.3%)
Maximum testing difference	95 (5.5%)	76 (6.8%)	87 (12.2%)	85 (6.3%)	85 (6.9%)	99 (7.5%)
Average testing difference	11.9 (1.2%)	10.5 (1.0%)	11.3 (1.1%)	11.6 (1.1%)	15.1 (1.5%)	11.7 (1.1%)
CPU time (s)	2.3	4.6	3.0	6.4	5.1	2.8

**Table 18:** The performance of ANN models with 2 different training datasets.

Performance	Random Pick	K-Medoids
Maximum training difference	0 (0)	0 (0)
Average training difference	0 (0)	0 (0)
Maximum testing difference	107 (16.5%)	76 (6.8%)
Average testing difference	12.4 (1.3%)	10.5 (1.0%)
≥50 cycles	205	66
≥7%	115	1
CPU time (s)	7.2	4.6

**Table 19:** The performance results of ensemble learning.

Performance	5 Sub-Models	15 Sub-Models
Maximum training difference	15 (2.2%)	12 (1.7%)
Average training difference	2.8 (0.3%)	1.9 (0.2%)
Maximum testing difference	66 (3.9%)	71 (4.2%)
Average testing difference	6.9 (0.7%)	7.0 (0.7%)
≥50 cycles	9	5
≥7%	1	1

**Table 20:** The hyperparameters of the sub-models.

Sub-Model	Preprocessing	Solver	Neuron Number
1	Robust scaler	L-BFGS	16-52-80
2	Robust scaler	Adam	76-28-84
3	Robust scaler	L-BFGS	72-24-8-80
4	Robust scaler	Adam	80-96-56-88
5	Robust scaler	L-BFGS	56-42
6	Robust scaler	Adam	72-96-88
7	Robust scaler	L-BFGS	60-84-68
8	Robust scaler	Adam	72-88-40-48
9	Robust scaler	L-BFGS	88-56-46-48
10	Robust scaler	L-BFGS	94-26
11	Robust scaler	L-BFGS	76-80-56
12	Standard scaler	L-BFGS	88-80-16-56
13	Standard scaler	L-BFGS	88-24-80-96
14	Standard scaler	L-BFGS	80-96-8-88
15	Standard scaler	Adam	80-64-16-8

**Table 21:** The final comparison results.

Item	Case I	Case II	Case III
Data selection	Random pick	K-medoids	K-medoids
Ensemble learning	No	No	Yes
Maximum training difference	0 (0)	0 (0)	12 (1.7%)
Average training difference	0 (0)	0 (0)	1.9 (0.2%)
Maximum testing difference	107 (16.5%)	76 (6.8%)	71 (4.2%)
Average testing difference	12.4 (1.3%)	10.5 (1.0%)	7.0 (0.7%)
≥50 cycles	205	66	5
≥7%	115	1	1

## Data Availability

Due to project privacy concerns, the data cannot be disclosed publicly.
